# Spatial-Temporal Patterns and Inflammatory Factors of Bone Matrix Remodeling

**DOI:** 10.1155/2021/4307961

**Published:** 2021-11-03

**Authors:** Jiechen Wang, Fengyuan Guo, Guangjin Chen, Jiwei Sun, Qingming Tang, Lili Chen

**Affiliations:** ^1^Department of Stomatology, Union Hospital, Tongji Medical College, Huazhong University of Science and Technology, Wuhan 430022, China; ^2^School of Stomatology, Tongji Medical College, Huazhong University of Science and Technology, Wuhan 430030, China; ^3^Hubei Province Key Laboratory of Oral and Maxillofacial Development and Regeneration, Wuhan 430022, China

## Abstract

The bone extracellular matrix (ECM) contains organic and mineral constituents. The establishment and degradation processes of ECM connect with spatial and temporal patterns, especially circadian rhythms in ECM. These patterns are responsible for the physical and biological characteristics of bone. The disturbances of the patterns disrupt bone matrix remodeling and cause diverse bone diseases, such as osteogenesis imperfecta (OI) and bone fracture. In addition, the main regulatory factors and inflammatory factors also follow circadian rhythms. Studies show that the circadian oscillations of these factors in bone ECM potentially influence the interactions between immune responses and bone formation. More importantly, mesenchymal stem cells (MSCs) within the specific microenvironments provide the regenerative potential for tissue remodeling. In this review, we summarize the advanced ECM spatial characteristics and the periodic patterns of bone ECM. Importantly, we focus on the intrinsic connections between the immunoinflammatory system and bone formation according to circadian rhythms of regulatory factors in bone ECM. And our research group emphasizes the multipotency of MSCs with their microenvironments. The advanced understandings of bone ECM formation patterns and MSCs contribute to providing optimal prevention and treatment strategies.

## 1. Introduction

Bone ECM is composed of organic constituents (25%), inorganic compounds (65%), and structural water (10%). The organic matrix principally comprises type I collagen and noncollagenous proteins. There are several kinds of noncollagenous proteins: *γ*-carboxyglutamic acid-containing proteins, glycoproteins, sialoproteins, proteoglycans, and enzymes [1]. They are mainly from osteoblasts before mineralization. The inorganic matrix is mainly poorly crystalline hydroxyapatite (HA) with minor trace elements [2]. These ingredients can transmit signals that influence adhesion, migration, proliferation, apoptosis, or differentiation by interacting with epithelial cells [3]. The dysregulation of bone ECM compositions destroys the balance of ECM remodeling and influences ossification.

Bone ECM is a highly dynamic structure that constantly undergoes controlled remodeling [3]. The architectures and temporal patterns play crucial roles in this process. Osteoblasts deposit matrix protein in lamellae with orientation alternating parallel or orthogonal for the primary stress axis of the structure, where the mineral matrix subsequently deposits on [4]. The mineral matrix matures from the amorphous ingredient to HA, which depends on matrix vesicles (MVs) and MV-like particles budded from osteoblasts to product phosphate and to remove protons liberated during calcium phosphate salt deposition. And they terminally are embedded in collagen fibrils by incipient interactions and subsequent constraints from the fibrils. Except for spatial orders, our research group tends to pay attention to temporal patterns of bone ECM. Recent studies show that the microcrystallization during mineralization is periodic [5]. Besides, type I collagen of ECM shows circadian rhythms in secretion and transportation processes, suggesting that the formation process of bone ECM likely shows circadian rhythms [6]. In addition, matrix metalloproteinases (MMPs) that can degrade the matrix are under the control of circadian rhythms [7]. These patterns maintain normal morphological structures and biological functions of bone. When these spatial and temporal patterns are disturbed, ECM remodeling is disorganized. This disorder reduces the rigidity and strength of bone, increasing the risk of bone fracture and other bone diseases.

Multiple regulatory factors and signaling pathways take part in regulating bone formation during bone ECM remodeling, particularly transforming growth factor *β* (TGF-*β*) signaling, bone morphogenetic protein (BMP) signaling, and inflammatory factors of the immune system [8]. Since these factors play significant roles in bone ECM formation patterns, they also can show circadian oscillations. Besides, studies have shown that the circadian clock controls immunoinflammatory responses [9]. Circadian rhythms potentially influence the interactions between immune responses and bone formation. And therefore, these inflammatory factors of the immune system or signaling pathways following circadian rhythms can serve as therapeutic targets for bone repair.

ECM and signaling factors make up the specific microenvironment of MSCs, which are crucially significant for tissue regeneration. Depending on their niches, bone marrow-resident MSCs possess multipotency and self-renewal ability. They can directly migrate to damaged tissue, proliferate, and differentiate into a wide range of tissue types [10]. Specifically, the paracrine function of the MSC-produced secretome is responsible for tissue regeneration, which connects with immunoregulation, anti-inflammatory effects, and angiogenesis [11]. And paracrine of MSCs is associated with extracellular vesicles (EVs). The progress of various scaffolds can induce the homing and differentiation of MSCs into specific lineages [12]. The interactions of MSCs and scaffolds connect with the physical properties and surface modifications of scaffolds [13]. And the scaffolds provide favorable microenvironments and adequate nutrition to promote angiogenesis and immunoregulation, which are interacted with tissue regeneration [14].

In this review, we summarize the formations of type I collagen and HA. Our research group reviews recent advances and provides new understandings of normal bone ECM remodeling concerning spatial and temporal patterns of ECM. We also analyze the disturbances of these patterns in pathological conditions. Besides, we pay attention to the circadian rhythms of inflammatory factors in bone ECM. Finally, we focus on the functions of MSCs within the multipotential microenvironments and biomaterials for tissue regeneration. These advanced understandings are possible to provide new therapeutic strategies for bone repair.

## 2. The Main Constituent Synthesis in Bone ECM Formation

### 2.1. Type I Collagen Self-Assembly Process

**Figure 1:**
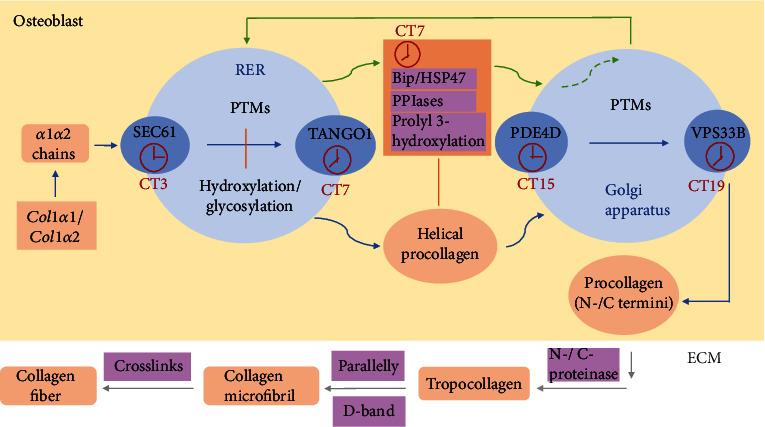
The rhythmic formation process of collagen. For the formation of collagen, *Col1α1* and *Col1α2* genes code *α*1 and *α*2 polypeptide chains severally. In the RER and Golgi apparatus, polypeptide chains fold into helical procollagen through posttranslational modifications, including hydroxylation and glycosylation. And molecular chaperones in this process like BiP, HSP47, PPIases, and prolyl 3-hydroxylation are responsible for the transport and correct conformation [6, 28]. What is more, the proteins for regulating collagen transport like SEC61, TANGO1, PDE4D, VPS33B, and BiP are under the control of circadian rhythms with different oscillations. When procollagen transports outside osteoblasts to form tropocollagen, its N- and C-termini are hydrolyzed [16, 18]. Tropocollagen deposits side by side and orients parallelly in one fibril but staggers with each other as the *D*-band. Eventually, multiple fibrils constitute collagen fibers with cross-links [19]. RER: rough endoplasmic reticulum; PTMs: posttranslational modifications; PPIases: peptidyl-prolyl *cis*-*trans* isomerases; ECM: extracellular matrix.

Type I collagen forms a triple helical structure with two identical *α*1 chains and one different *α*2 chain [15]. In osteoblasts, the ribosomes on the rough endoplasmic reticulum (RER) code *α*1 and *α*2 polypeptide chains severally following *Col1α1* and *Col1α2* genes. And then, polypeptide chains are transported in the forms of procollagen molecules with signal peptides for recognition. Signal peptides are excised once procollagen molecules get into the ER. Proper chain recognition and heterotrimer assembly in ER depend on the interactions with ER-resident molecular chaperones, including Serpinh1 (HSP47), binding immunoglobulin protein (BiP), the prolyl 3-hydroxylation (*CRTAP*, *LEPRE1*, and *PPIB*) complex, and peptidyl-prolyl cis-trans isomerases (PPIases) [16]. Hydroxylation of proline benefits for hydrogen to bond with water and other amino acids within the collagen chains. Besides, glycosylation and hydroxylation of lysine are responsible for cross-link formation, which can later transform to mature enzymatic cross-links for the stability of the self-aligned collagen molecules (Figure 1) [17]. With the help of heat shock proteins, procollagen molecules enter into the Golgi apparatus for further modifications. These posttranslational modifications (PTMs) are vital for the overall structures and mechanical properties of bone.

Procollagen molecules contain small terminal globular prodomains known as the amino (N-) and carboxyl (C-) termini. When procollagen molecules translate into tropocollagen molecules, the proteinases hydrolyze the termini into terminal telopeptides [16, 18]. These collagen molecules orient parallelly on the *c*-axis in one microfibril. There is a partial overlap among these molecules. It produces an apparent periodicity known as the *D*-band where *D* = 67 nm, including the gap zone with a straight channel and the overlap zone [19]. In addition, the collagen molecules closely accumulate by the quasi-hexagonal way [20]. The analysis observes that unmineralized collagen fibrils organize in a triclinic superstructure, comprising microfibrils with only a degree of lateral organization [4]. Besides, each microfibril includes five 1D staggered, twisted collagen triple helix molecules [4] (Figure 1).

### 2.2. Two Mineralization Mechanisms in ECM

Increasing evidence supports that MVs and MV-like particles mediate mineral mineralization. They are diminutive membrane particles budded off from the plasma membrane of chondrocytes, osteoblasts, and odontoblasts [21]. The transmission electron micrograph shows collagen fibrils are adjacent to MVs with crystals forming. Besides, there is a space between osteoblasts and MVs, where has a lot of ions like Ca^2+^ and PO_4_^3−^. These ions are regulated to form HA and maintain homeostasis by Ca^2+^ and PO_4_^3−^ ion pumps of MVs. Intracellular nucleoside triphosphate (iNTP) transforms into the intracellular Pi (iPi) by adenosine triphosphatase (ATPase), and the intracellular pyrophosphate pool (iPPi) also contributes to iPi by intracellular pyrophosphatase (iPPase). Then, iPPi is secreted out of MVs by ankylosis protein (ANK), and iNTP transports into ECM as extracellular PPi (ePPi). And iNTP also could transform into iPPi by nucleotide pyrophosphatase phosphodiesterase 1 (NPP1). Extracellular PPi and iNTP hydrolyze to Pi via tissue nonspecific alkaline phosphatase (TNAP). In the meantime, intracellular Ca^2+^ and iPi also translate into the matrix. Pi and Ca^2+^ synthesize HA deposited in collagen fibrils [21]. On the other hand, Ca^2+^ and PO_4_^3−^ are transported into MVs through annexin channels and the type III Na^+^/PO_4_^3−^ cotransporters Pit1,2 for homeostasis.

**Figure 2:**
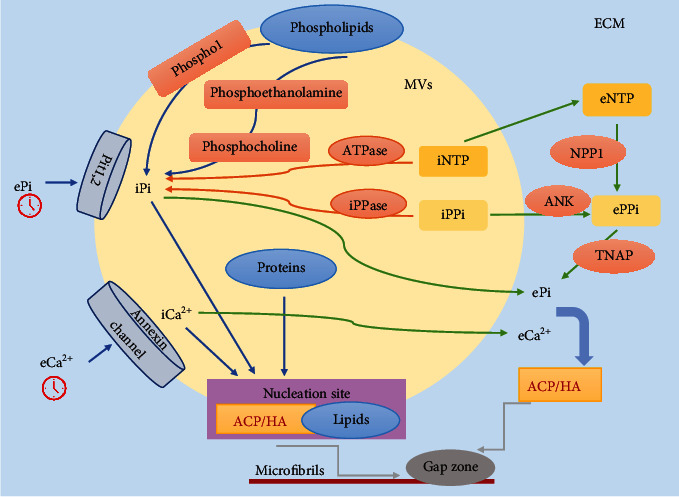
The two patterns of ACP/HA formation by MVs. For one pattern, iNTP transforms into iPi through ATPase, and iPPi transforms into iPi by iPPase. Then, iPPi is secreted out of MVs by ANK, and iNTP transports into ECM as ePPi. And iNTP could transform into iPPi by NPP1. Extracellular PPi and iNTP hydrolyze to Pi via TNAP [21]. In the meantime, intracellular Ca^2+^ and iPi translate into the matrix. ePi and eCa^2+^ synthesize HA or ACP. On the other hand, PHOSPHO1 activates the formation of apatite crystals by hydrolyzing phospholipid and collaborating with phosphoethanolamine and phosphocholine [23]. The annexin channels allow a mass of Ca^2+^ to enter into MVs [24]. It is possible that lipids in the membranes of MVs are nucleation sites, and Ca^2+^, Pi, and some other proteins in MVs constitute specific structures, where HA or ACP could crystallize [25]. MVs: matrix vesicles; iNTP: intracellular nucleoside triphosphate; ATPase: adenosine triphosphatase; iPPi: intracellular pyrophosphate pool; iPPase: intracellular pyrophosphatase; ANK: ankylosis protein; eNTP: extracellular nucleoside triphosphate; NPP1: nucleotide pyrophosphatase phosphodiesterase 1; ePPi: extracellular pyrophosphate pool; TNAP: tissue nonspecific alkaline phosphatase; ACP: amorphous calcium phosphate; HA: hydroxyapatite; ePi: extracellular Pi; iPi: intracellular Pi; eCa^2+^: extracellular Ca^2+^; iCa^2+^: intracellular Ca^2+^.

However, it has been observed that mineral crystals are synthesized in MVs in alkaline phosphatase-deficient mice [22], suggesting that there is another mechanism of mineralization. PHOSPHO1, a matrix vesicle membrane-associated phosphatase, collaborates with phosphoethanolamine and phosphocholine to activate the formation of apatite crystal by hydrolyzing phospholipid on the matrix vesicle membranes [23]. According to the studies, the membranes of MVs are specifically imbued with phosphatidylserine (PS) and acid phospholipids, combining annexins and allowing a mass of Ca^2+^ to enter into MVs [24]. Lipids in the membrane of MVs may be nucleation sites, and Ca^2+^, Pi, and some other proteins in MVs constitute specific structures, where HA crystals could crystallize [25], whereafter the mineral crystals enter into collagen fibrils and further grow (Figure 2).

## 3. The Spatial Structures and Temporal Patterns of Bone ECM Remodeling

Type I collagen and inorganic minerals constitute the basic structural framework of bone ECM. Since bone ECM is highly dynamic and undergoes controlled remodeling, the formation and degradation of these compositions are probably regular. The regular changes are responsible for ECM structure, stiffness, and biological functions. Besides, these order patterns can contribute to the studies of bone diseases and the developments of personalized precision therapies.

### 3.1. The Periodicity and Circadian Rhythms Implement Precise Regulations

Type I collagen plays an important role in osteoblast attachment, proliferation, and differentiation, providing osteogenesis. It provides the scaffolds for osteoblast-lineage cells and mineral crystals. Additionally, proper collagen formation and organization are associated with strength, postyield strains, and fracture toughness [26, 27]. Recent studies confirm that circadian rhythms in the selective mechanisms of protein homeostasis maintain ECM structure and function. Chang et al. prove that the transport of procollagen-I (PC-I) by the collagen protein secretory pathways in fibroblasts is under the control of the circadian clocks. The proteins that, respectively, control the entrances and exits of the ER and Golgi are 24 h rhythmic, including SEC61, TANGO1, PDE4D, and VPS33B [6, 28]. In addition, Pickard et al. show that BiP is under the control of the circadian clocks, which take part in the collagen secretion process and assist collagen folding (Figure 1) [29]. In the Clock^*Δ*19^ mice with a defective circadian clock, the numbers of collagen and the structure of fibrils are abnormal. It also decreases elastic modulus and maximum load [28]. At another pathological state, misfolded collagen chains in the ER activate the unfolded protein response (UPR) in OI with the help of chaperone increases or mutant protein degradation [30]. Glycine substitutions in OI postpone collagen folding, causing overmodified collagen and probably damaging the secretion process [31]. The overmodified normal collagen proteins in recessive OI increase the risks of the direct impact on account of exorbitant hydroxylation and glycosylation in the extracellular matrix. One research studies several bone biomarkers in women exposed to the incorporation of sleep restriction with circadian disruption (SRCD). The preliminary data from these women show that the contents of N-terminal propeptide of type I procollagen decrease in women, and the contents of C-telopeptide increase in the young. Another study demonstrates a rapid suppression of N-terminal propeptide in type I procollagen in men with SRCD [32]. These two findings indicate that the dysregulations of circadian rhythms destroy the collagen formation patterns and inhibit bone formation [33].

Studies demonstrate that the stiffness, fracture strength, and robustness of bone and type I collagen are enhanced when HA crystals efficiently bear the stress applied to the collagen. The microcrystallization during mineralization is periodic by transmission electron microscopy, with the pH-dependent characters occurring periodically in collagen [5]. A time-resolved study reveals that calcium phosphate particles are present outside the fibrils after 24 h. These particles approach the gap zone and show a diffuse band characteristic of ACP. Then, HA crystals begin to synthesize in ACP after 48 h. And after 72 h, extended electron-dense crystals collect in the fibrils, which generally inlay in a less dense matrix. It suggests that the establishment of bone ECM possibly follows periodicity. Generally, the formation of minerals in the extracellular matrix depends on Ca and P metabolism. Plasma content of Ca presents 24 h rhythmic changes with a diurnal peak, and plasma P is reported to follow circadian rhythms with a peak in the evening [34]. Enamel is the most mineralized tissue secreted from ameloblast cells. Enamel crystallites form enamel prisms, which show cross-striations through polarized light. And these cross-striations are connected with daily via scanning electron microscopy [35]. The *Amelx* that serves as a differentiation-specific product shows rhythmic oscillations in ameloblasts. Besides, other gene expressions of the mineralized enamel are also rhythmic, like *Nbce1* and *Car2*. It suggests that the synthesis and mineralization of enamel are related to circadian rhythms [35, 36]. A disrupted pattern of incremental lines in enamel is found in rats whose suprachiasmatic nucleus are excised, which also implies the relation of the circadian clocks [37]. Circadian rhythms are also observed in other mineralized matrices like the dentin matrix of rats by rhythmic lines from hematoxylin, toluidine blue, and silver nitrate staining. It forms successive microscopic growth lines inside tooth crowns and roots [38]. The radioautography studies show that the combination of ^3^H-proline and odontoblasts follows circadian rhythms. Proline is the significant amino acid of collagen and the noncollagenous dentin matrix, implying that circadian rhythms appear in collagen secretion [39].

Moya et al. prove that the light calcification rate is about 2.3 times higher than the dark, and there is a lag phase of calcification from day to night or from night to day when amino acid precursors transport into the organic matrix [40]. In addition, the animal models exposed to the mood-stabilizing drug valproic acid have sleep problems. It is probably because of the altered expressions of circadian clock genes. Simultaneously, differential expressions of collagen-encoding genes are likely to induce disturbances in extracellular matrix signaling. And this process is reported to be related to altered cortical development [41]. These two findings suggest that the mineralization process from minerals to collagen may conform to circadian rhythms.

MMPs act more effectively in bone ECM degradation. Physiologically relevant temperature oscillations alter the activities of MMP-2 and MMP-9. The activity of MMP-9 is suppressed by inhibiting *heat shock transcription factor 1* (*BMAL1*), which is responsible for peripheral circadian rhythms [42]. Additionally, MMP-9 activity improves after mild treadmill exercise, and the expression of tissue inhibitor of metalloproteinase-1 (TIMP-1) is invariant, suggesting that exercise triggers MMP-9 activation in the hippocampus [43]. Osteoarthritic changes are promoted in male mice with a disordered light-dark (LD) cycle due to the observable increases of MMPs and the decreases of anabolic mediators [7]. Furthermore, the physical location and the time frame of MMP enzymatic activity are fundamental to the physiological roles in tumor progression [44–47]. MMP-9 plays a specific role in the tumor vasculature, apoptosis, inflammation, and growth signals [47]. Additionally, pathological fibrosis also relates to cancers [48]. Therefore, the MMP activity patterns in matrix remodeling even affect the system homeostasis.

### 3.2. The Step-by-Step Integration of Components Follows the Stipulated Orders

In all bone diseases, bone fracture is a serious clinical problem. The proper integration of bone ECM components provides solid support for bone stuffiness. The disorders of ECM architecture will improve bone fragility and increase the risk of bone fracture [49]. Nowadays, biomaterials in bone regeneration also focus on the bone mineralization degree, hydroxyapatite crystal size, and precise microarchitecture of bone ECM, such as calcium phosphate-based ceramics [50]. In the biomimetic designs, these synthetic bone graft substitutes show similar or even closer performances by the better understanding of bone ECM formation patterns.

**Figure 3:**
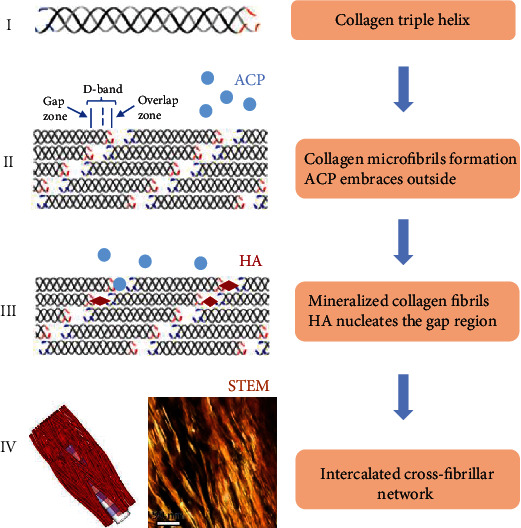
The quaternary hierarchical architecture of collagen and mineral assembly. The first structure is collagen molecules with the triple helix [15]. Secondly, collagen molecules form microfibrils with *D*-bands, and ACP embraces outside of microfibrils [54, 55]. Then, HA crystals nucleate the gap regions within mineralized collagen fibrils [55]. Finally, mineralized collagen fibers with mineral crystals create an intercalated cross-fibrillar network [59].

The organic matrix and minerals are assembled as fractal-like hierarchical architectures in a bottom-up manner [51, 52]. Concerning the establishment of bone ECM, it also follows a hierarchical pattern (Figure 3). Existing evidence states that the organic matrix of bone gives top priority to assembling and precisely regulates the mineral crystal nucleation and growth [53]. Amorphous calcium phosphate (ACP) transforms into the HA crystals, which nucleate the gap regions within the collagen fibrils both in vivo and in vitro [54, 55]. The first HA crystals are needle-like, which depends on channel shapes. Collagen fibrils control intrafibrillar nucleation pathways with a lessened nucleation energy barrier [56]. ACP-pAsp (polyaspartic acid) has a negative surface charge, and the entry sites have positive net charges and own the lowest electrostatic potential energy. The interaction of the positive and negative charges crucially propels ACP into the fibrils [55]. Furthermore, these crystals that grow unremittingly along with the *c*-axis of fibrils will extend to the overlap region, resulting in a rearrangement of the collagen molecules. It is coincident with SAXS/WAXS data on CaCO_3_. Ultimately, the needle-like crystals bend into flexuous HA platelets along with *c*-axes or even merge into small stacks.

Apatite crystals follow an asymmetrical, subtly splaying organization pattern inside the continuous collagenous matrix. HA crystals orient along with *c*-axes of microfibrils in an additional diffraction ring at ~2.8 Å, resulting in beyond recognition and diffractions [57]. These diffractions come from multiple lattice planes. They are concomitant when the HA platelet planes are misoriented by >60°, suggesting that the HA platelets are uniaxially oriented. And it also deduces an angular distribution of ~±20° in these orientations by tomographic construction of 150 crystals. Other crystal orientations can only grow via pushing the collagen molecules, which presumes that the HA crystal orientations depend on the restraints from collagen, not on the chemical interaction [57]. For extra fibrillar mineralization, the crystals aggregate and densify prenucleation clusters to form the spherical ACP, which is the intermediate product during the nucleation pathway. And the mineral crystals assemble in the unconfined space without a specific orientation [58]. The acicular mineral particles approximately connect with extra collagen fibrils and neighboring collagen fibrils, building an intercalated cross-fibrillar network [59]. Studies show that the density and orientation of ECM fibers control immune cell migration. Loose areas of fibronectin and collagen promote T cell motility, which potentially influences bone tissue remodeling [3].

Generally, the uniform gaps between the platy structures contain noncollagenous proteins, polysaccharides, disordered calcium phosphate, and structural water [60]. These elements are beneficial for flexibility and toughness through keeping a high aspect ratio of the single crystals. Structural water is an interfacial agent between collagen and HA. It interacts intensively with the inorganic mineral when a disordered layer of minerals covers the crystal nucleus. According to the advanced solid-state nuclear magnetic resonance (ssNMR) experiment, the disordered layer of minerals consists of Ca^2+^, HPO_4_^2−^, CO_3_^2−^, and water, with stronger hydrophilicity. This structure has the buffer capacity and provides a chemical environment for ion exchange, permitting mediating the acidity of the reaction medium under physiological conditions [61]. Besides, van der Waals attractive forces induced by structural water improve the interaction between the mineral platelets, causing the local stacking and orientation of the apatite platelets. Citrate and a number of noncollagenous proteins participate intrafibrillar mineralization by assisting mineral infiltration. And they are proposed to play specific roles in extra fibrillar mineralization, accounting for the mineral content in bone [55, 62, 63].

Bone ECM formation, organization, orientations, and chemical modifications provide physical support and felicitously mediate rigidity [45]. Additionally, structural integrity affects the morphology and biological function of the skeleton by regulating the behaviors of osteoblasts and osteoclasts. It emphasizes that the spatial architecture and periodic patterns of ECM remodeling are responsible for osteogenesis. And the advanced understanding should be studied further for new therapies of bone repair.

## 4. Regulatory Factors with Circadian Rhythms in Bone ECM

It indicates that circadian rhythms regulate bone ECM formation and remodeling by directly influencing ECM component expressions. Besides, bone ECM is imbued with regulatory factors that are released during remodeling. These factors play critical roles in mediating collagen and inorganic matters, such as TGF-*β*, BMPs, and inflammatory factors [64]. It potentially indicates that these regulatory factors also can be rhythmic. Thus, circadian clocks may be new therapeutic strategies of bone defect repair via regulating the regulatory factors.

### 4.1. Two Main Regulatory Factors Potentially Show Circadian Oscillations in Bone ECM Remodeling

**Figure 4:**
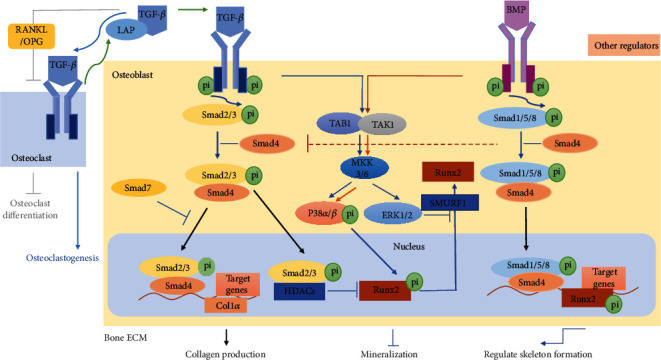
The main regulatory pathways of TGF-*β* and BMP in bone ECM. TGF-*β* is produced predominantly by osteoblasts and deposited into the matrix with LAP. Activated TGF-*β* requires osteoclastic bone resorption by dislodging LAP [67]. Smad2/3 is phosphorylated and connects with Smad4 when TGF-*β* combines with its receptors. The complex of Smad2/3 with Smad4 could transport into the upstream of *Col1α* and into other target gene transcription start sites. The complex upregulates gene expressions to promote collagen production [68, 69], and it is inhibited by Smad7. The phosphorylated Smad2/3 could inhibit the expression of Runx2 by combining with HDACs [70]. On the other hand, TGF-*β* influences Runx2 expression to suppress bone mineralization and osteogenesis by the MAPK pathway, independent of Smad3. The p38 MAPK pathway directly regulates the expression of Runx2. And the MAPK-ERK pathway reduces the expression of Runx2 by upregulating SMAD ubiquitination regulatory factor 1 (SMURF1) [71, 72]. BMP could improve the expression of Runx2 by Smad1/5/8 and Smad4 or the p38 MAPK pathway for regulating skeleton formation [75, 76]. And the process of the combination of Smad4 could suppress the formation of the Smad2/Smad3/Smad4 complex. TGF-*β*: transforming growth factor *β*; HDACs: histone deacetylases; ERK1/2: extracellular signal-regulated kinase 1/2; MKK: mitogen-activated protein kinase kinase; TAK1: transforming growth factor-activated kinase 1; SMURF1: SMAD ubiquitination regulatory factor 1; BMP: bone morphogenetic protein; LAP: latency-associated protein; RANKL: receptor activator of nuclear factor kappa B ligand.

TGF-*β* is a particular inducer among the cytokines both in vitro and in vivo [65, 66], which is produced predominantly by osteoblasts and deposited into the bone matrix with latency-associated protein (LAP). Activated TGF-*β* requires osteoclastic bone resorption by dislodging LAP [67]. TGF-*β* can downregulate the RANKL/OPG secretion ratio to inhibit osteoclast differentiation, although it can improve osteoclastogenesis by connecting with its receptors on osteoclasts [8]. TGF-*β* participates in promoting collagen formation by increasing the expressions of *Col1α1* and *Col1α2* [68]. Kahai et al. have observed that *COL5A1* (*α1*) expression increases when MC3T3-E1 cells interact with TGF-*β*1 by Northern blotting. TGF-*β* signaling targeting COL5A1 shows osteogenic potential by treatments in vitro and in vivo. It also has found that the TGF-*β* response element (T*β*RE) exists in the upstream of the *Col1α1* transcription start site in mice, and it could activate *Col1α* with the assistance of various receptor complexes, such as Smad2/Smad3/Smad4 [68, 69]. And Smad7 inhibits the activity of the Smad2/Smad3/Smad4 complex (Figure 4). Further studies report that Runx2 is an essential target of TGF-*β*/Smad3 in modulating bone ECM quality. TGF-*β*/Smad3 suppresses Runx2 by directly interacting with Runx2 at Runx2-binding DNA sequences of osteoblast differentiation genes. The inhibition of Runx2 leads to transcription repression at the osteocalcin promoters. In this process, histone deacetylases (HDACs) are recruited to cell nuclei in the control of the Runx2/Smad3 complex, forming a stable complex of Smad3, Runx2, and HDAC at the Runx2-binding DNA sequences. Bone ECM is hypomineralized with decreased hardness and elastic modulus in HDAC3-deficient mice. This mechanism contrasts the repression of myogenic transcription by TGF-*β*/Smad3, which does not involve HDAC recruitment [70]. Studies show that TGF-*β* influences Runx2 expression to suppress bone mineralization and osteogenesis by the MAPK signaling pathway, independent of Smad3 [71, 72]. The classical MAPKs include extracellular signal-regulated kinase 1/2 (ERK1/2), p38 kinases, c-Jun N-terminal kinases (JNKs). Phosphorylated transforming growth factor-activated kinase 1 (TAK1) recruits transforming growth factor *β*-activated kinase-binding protein 1 to initiate the mitogen-activated protein kinase kinase- (MKK-) p38 mitogen-activated protein kinase (p38 MAPK) or MKK-ERK1/2 signaling pathways when TGF-*β* connects to its receptors. Then, the p38 MAPK pathway directly regulates the expression of Runx2. The MAPK-ERK pathway reduces the expression of Runx2 by upregulating the expression of SMAD ubiquitination regulatory factor 1 (SMURF1), which is a significant ubiquitin ligase to mediate proteasomal degradation of Runx2 (Figure 4) [8, 72]. In short, TGF-*β* serves as a critical regulatory factor of the bone ECM regulation network. It demonstrates that *BMAL1* is disrupted in human OA cartilage. And a loss of *BMAL1* reduces the phosphorylated Smad2/3 [73]. Besides, *BMAL1* promotes TGF-*β*1-induced profibrotic activities in the fibroblasts, and the activation of TGF-*β*1 improves the transcriptional induction of *BMAL1* [74]. These results show that TGF-*β* is controlled by circadian genes. And more studies should focus on the interactions of TGF-*β* and circadian rhythms in bone ECM.

BMPs are members of the TGF-*β* superfamily. BMPs can inhibit TGF-*β*-mediated fibrotic gene expressions by activating Smad1/5/8 [75]. The interactions of Runx2 and BMP/TGF-*β*-activated Smads are critical for osteogenesis [76]. BMP6^−/−^ fibroblasts show more intensive collagen contraction than the control cells through a free-floating collagen contraction assay, and the expression of *Col1α1* improves in BMP6^−/−^ fibroblasts, which is associated with AP-1. Elevated expressions of AP-1 family member c-Jun can accelerate the production of the extracellular matrix, and BMP6 recognizes it as a negative regulator of AP-1 activity [77]. And it is reported that the combination of BMP2 and vascular endothelial growth factor (VEGF) benefits both bone mineralization and the expression of the organic matrix. A short-term BMP2 expression is indispensable to induce bone formation. The up-regulation of BMP2 extremely increases osteocalcin [78, 79]. BMP7 induces the expression of osteoblastic differentiation markers and promotes calcium mineralization (Figure 4) [75, 80]. These studies show that BMPs are responsible for ECM remodeling and bone formation. Researches then demonstrate that *Bmp2* and *Bmp6* mRNA levels coincide with melatonin levels that follow circadian rhythms [81]. Min et al. observe that BMP2 expression is attenuated in *Bmal1* knockout (KO) cells. And in MC3T3-E1 cells, Bmal1 activates osteoblast differentiation by the regulation of BMP2 [82]. More importantly, advanced therapies can be applied to promote bone defect repair by improving BMP2 expression following circadian rhythms.

### 4.2. Immunoinflammatory Systems Regulate Bone Formation following Circadian Rhythms

Inflammatory factors play crucial roles in ECM remodeling and osteogenesis. And studies have shown that circadian oscillations of immune mediators coincide with the activity of the immune system, indicating that the cooperation of the circadian clocks and the immune system potentially regulates bone formation.

Interleukins (ILs) take part in regulating collagen formation. IL-1*β* effectively reduces the expression of collagen mRNA by selectively increasing the EP_4_ receptor in the p38 MAPK signaling pathway [83]. And proteins combined with IL-1*α* could interact with HAX-1 and IL-1 II receptors to promote the expression of collagen. IL-1 expression shows circadian rhythms, and the peak expression occurs during the night and early morning [84]. IL-6 could also increase the expression of collagen [85]. Besides, IL-6 exhibits both membrane-bound and soluble signaling in osteoblasts and osteocytes, supporting the development of bone-resorbing osteoclasts by acting early in the osteoblast lineage [86]. Studies show that IL-6 has circadian oscillations [87]. The serum level of IL-6 significantly increases by stimulating the toll-like receptor 4 (TLR4) at the beginning of the active phase [88].

Interferons (IFNs) and tumor necrosis factor *γ* (TNF-*γ*) have crucial effects on regulating the immune responses and modulating the dynamic balance of bone ECM [89]. IFN-*α* can activate osteoblast differentiation and inhibit osteoclast fusion to maintain bone ECM integrity. It shows that the expressions of IFN-*α* and TNF-*γ* follow circadian rhythms. And an earlier and higher peak of TNF-*γ* is found in rats with collagen-induced arthritis [84]. REV-ERBs constitute a negative feedback loop of the circadian clock genes. The loop inhibits a part of inflammatory genes in a signal-dependent manner, dictating temporal expressions of inflammatory genes. The mechanism is that REV-ERBs can mediate repression by recruiting of NCoR complexes of HDAC3 [9]. These results indicate that the circadian clocks can be recognized as a bridge between the immune system and bone formation.

Immunoinflammatory responses play significant roles in osteogenesis. And infection is a crucial factor in preventing bone formation. Cyclical exposure to inflammatory injury or other pathogens matches the expression oscillations of pattern recognition receptors (PPRs) on tissue macrophages, leading to the periodical release of IL-6 and TNF. These factors activate systemic inflammatory responses, causing the rhythmic release of leukocytes and the rhythmic recruitment of leukocytes to tissues. A rhythmic interaction of leukocytes and endothelial cells inhibits blood flow in small-caliber vessels, obstructing the bone tissue vascularization during bone formation [90]. Therefore, new therapies of bone defect repair can focus on the circadian rhythms of inflammatory factors. It will promote collagen formation or improve biomineralization. Besides, it is responsible for promoting bone formation by anti-infection and vascular remodeling.

## 5. Stem Cells and Relevant Biomaterials in Tissue Regeneration

In addition to spatial-temporal patterns of ECM, resident stem cells of bone also play crucial roles in regenerative medicine. Compared with extraosseous MSCs, bone marrow-resident MSCs and hematopoietic stem cells (HSCs) are assumed to reside within a specialized microenvironment or niche regulated by a combination of local and systemic effects [91]. The specific niches show multipotency, controlling MSC behaviors and maintaining tissue homeostasis [14]. Recently, studies and clinical trials pay attention to the biomaterials that provide stem cells with a sustaining and favorable microenvironment for increasing regeneration potential.

### 5.1. Resident MSCs with a Specific Microenvironment Show Multipotential Properties

Resident MSCs possess multipotency and self-renewal ability. In physiological conditions, they are at rest within the specific niches. These niches consist of ECM, cells, and abundant neurovascular bundles. And they are regulated by the circulatory microenvironment including inflammatory factors, metabolites, hormones, and other soluble factors [92]. Importantly, the MSC-derived secretome is able to recapitulate crucial properties of MSCs [11]. MSC secretome contains chemokines, growth factors, cytokines, and immunomodulatory molecules. It can promote tissue regeneration by inducing tissue remolding, regulating immune response, stimulating vascularization, and inhibiting fibrosis [93]. However, excessive pathological conditions can change the specific niches, leading to the influences of stem cell properties and the damage of tissues.

In response to injury signals, MSCs potentially move from their niches into the target tissues. This complicated homing process is regulated by chemical factors and mechanical factors. Studies show that the stromal cell-derived factor-1 (SDF-1)/CXC chemokine receptor 4 (CXCR4) axis assists MSC migration. The SDF-1 remarkably increases after tissue injury. When the SDF-1 concentration is lower than 100 ng/m, increased SDF-1 upregulates the number of migrated MSCs [94]. And the expression of CXCR4 also increases. It has been confirmed that the up-regulation of CXCR4 increases the transplant of BMSCs in infarcted myocardium [95]. In addition, OPN is a crucial regulator. Studies reveal that OPN reduces organized actin cytoskeletons through the ERK and FAK pathways to promote MSC migration [96]. Other regulatory factors such as TGF-*β* and basic fibroblast growth factor (bFGF) also play essential roles. With regard to mechanical factors, the MSC homing process is regulated by stiffness, mechanical strain, microgravity, and shear stress [97]. After division and proliferation, MSCs directionally and multidirectionally differentiate into specific cells for tissue regeneration through the pathological microenvironment. In bone tissue regeneration, the BMP signal phosphorylates a series of downstream proteins like Smad1/5/8 and TAK1 by cognate receptor serine/threonine kinases, causing osteoblast differentiation [98]. Insulin-like growth factor-1 (IGF-1) that is responsible for osteoblast differentiation can be modulated by IGF-binding proteins (IGFBPs) [99]. Runx2 phosphorylated by ERK1/2 and p38 MAPK regulates osteoblast differentiation [100]. Besides, Osterix and ATF4 are also osteoblastic transcription factors [101, 102]. In vivo, osteoblastic differentiation of MSCs commonly connects with the downregulation of adipocytic differentiation [103].

In addition to direct differentiation, MSCs repair the impaired tissue through paracrine. Rat BMSCs secrete paracrine factors such as FGF-2, TGF-*β*, vascular endothelial growth factor (VEGF-1), and angiopoietin-2 to active angiogenesis at injury sites, promoting myocardial repair [104]. In rats with skin wounds, the stromal cell-derived factor-1- (SDF-1-) engineered MSCs (SDF-MSCs) secrete IL-6, hepatocyte growth factor (HGF), VEGF to promote the wound healing activity of MSCs [105]. Importantly, the paracrine of MSCs is associated with their anti-inflammatory and immunosuppressive properties [106]. In acute kidney injury, the protective action of MSC is due to enhanced regulation of anti-inflammatory factors like IL-10 [107]. MSCs potentially alter the macrophage M1 phenotype to the M2 phenotype, increasing IL-10 secretion and reducing TNF-*α* production. It regulates the inflammatory response to bacterial injury [108]. Studies show that hMSCs change the cytokine secretion profile of T cells, dendritic cells (DCs), and natural killer (NK) cells to develop immunologic tolerance. The secretion of TNF-*α* decreases from DC1 by hMSCs, and the secretion of IL-10 increases from DC2 by hMSCs. IL-4 secretion increases from Th2 cells, and IFN-*γ* decreases from NK cells via hMSCs. It may relate to the increased expression of prostaglandin E2 (PGE2) from hMSCs [109]. Furthermore, hMSCs inhibit proliferation and alloreactivity of T cells, which is related to the enhanced galectin-1. And MSC-derived galectin-1 regulates cytokine release like graft-versus-host disease (GVHD) and anti-inflammatory factors such as IL-2 and IL-10 [110].

It has been confirmed that the paracrine of MSCs is associated with EVs. EVs are primarily released from the endosomal compartment and comprise proteins, miRNA, mRNA, long noncoding RNAs, and phospholipids [111]. Generally, EVs have common proteins, including TSG101, tetraspanins (CD81, CD63, and CD9), and Alix. And EVs also comprise specific proteins that reflect the source and the pathophysiological states of the cell source [112]. Researches study the proteomic signature of MSC-EVs and show that MSC-EV proteins containing markers of MSCs can modulate the self-renewal and differentiation properties of MSCs [113]. Besides, the functional analysis reveals these proteins take part in cell commutation, biogenesis, inflammation, and motility of EVs [114]. And they play positive roles in specific diseases. It has been observed that cardiac remodeling is regulated by miR-22-loaded EVs by targeting methyl-CpG-binding protein 2 [115]. Furthermore, five enzymes that take part in the ATP-generating stage of glycolysis are associated with MSC-mediated alleviation of myocardial damage [116]. Hypoxia leads to cell adaptation to low oxygen by the upregulation of proteins including anaerobic metabolism, autophagy, and cell migration [117].

These studies indicate that resident MSCs show great potential in tissue regeneration. The MSC-produced secretome is responsible for this property, which concerns multiple aspects of regulation. And therefore, the healing strategy targeting pathological microenvironments including the MSC-produced secretome and MSC-EVs can promote MSC therapeutic potential. Although a wide range of regulatory factors has been studied, there are still many limitations to be worked out. For instance, most results are found in vitro and ignore the synergistic effects of multiple mechanical and chemical factors. In addition, the specific signaling pathways and internal mechanisms of these regulation processes need further discussion.

### 5.2. The Interactions of Stem Cells and Biomaterials Provide Advanced Strategies for Regeneration Medicine

Accordingly, the multipotential microenvironment plays a critical role in controlling the regeneration property of endogenous and exogenous stem cells. On the one hand, the treatment strategy focuses on repairing the pathological microenvironment for improving the resident stem cell-based regeneration. On the other hand, promoting the resistance of stem cells to the diseased microenvironment accelerates the efficacy of transplanted stem cells [14].

Interestingly, biomaterial-based scaffolds provide a suitable microenvironment for stem cells and promote stem cell adhesion, growth, and differentiation. Numerous requirements and prominent advantages should be taken into consideration when selecting a siutable scaffold for the proliferation and differentiation of stem cells. Naturally derived polymeric scaffolds have been extensively applied in biomaterial applications. Hyaluronic acid (HA) comprises alternating D-glucuronic acid and N-acetyl-D-glucosamine units, inducing cellular migration by activating signaling pathways [118]. Besides, the porous architecture of HA and its derivatives allow solute diffusion and nutrient/waste exchange. Limitations of HA-based scaffolds principally contain the requirement to be linked with growth factors like BMP2. And it may supervene with hypersensitivity reactions [119]. The chitosan scaffold serves as a functional delivery aid to support the platelet lysate, stem cells, and growth factors. It has been demonstrated that insufficient vascularization from the biomaterials will interfere with bone regeneration in clinical trials. The vascularization process involves endothelial cells and growth factors in different stages, including VEGF, angiopoietin, and FGF [120]. A ploylactic acid (PLA) foam contains chitosan-chondroitin sulfate nanoparticles loaded with the platelet lysate. The platelet lysate provides multiple growth factors such as FGF, platelet-derived growth factor (PDGF), and TGF-*β* for vascularization and osteogenic differentiation of MSCs [121]. And the porosity of the chitosan-based scaffold potentially provides proper gaps between stem cells and blood vessels. In clinical trials, the scaffold that supports uniformly distributed stem cells provides an advantageous microenvironment for intensive viability and potential angiogenesis [120]. However, the complicated gelation and degradation mechanisms of chitosan limit its applications in the injectable scaffold [122]. Accordingly, the microenvironment consisting of neighboring cells, ECM, and plentiful neurovascular bundles regulates the biological behaviors of stem cells, which shows multipotency with stem cells. Therefore, some naturally derived biomaterials such as native ECM scaffolds and skeletal muscle acellular scaffolds (MAS) display potential for development. Aulino et al. demonstrate that MAS mimics a multipotent environment that allows the homing of stem cells and their multidirectional differentiation toward different cell lineages through microenvironmental signals. It may provide a new technique for complex regeneration processes like the regeneration of musculoskeletal tissues [123].

As for synthetic scaffolds, poly(L-lactic acid) (PLLA) is one of the few synthetic degradable polymers that can be applied in clinical trials [124]. It is a biomaterial with nanoscale polymer fibers, which is produced by electrospinning. The combination of nanofibers and a rotating mandrel can mimic the anisotropic morphology of some tissues [125]. The study has observed that the electrospun PLLA/5% lecithin scaffolds can maintain the phenotypic shape of MSCs and integrate MSCs with the microfibers [126]. However, the acidic residues from the degradation of PLLA will suppress local cell activity. Tatullo et al. add calcium silicate (CaSi) and dicalcium phosphate dihydrate (DCPD) into PLA to improve the biointeractivity, biocompatibility, and mechanical properties. They also observe that the homogeneously distributed mineral compounds play crucial roles in the homogeneous bioactivation of the cells along the whole scaffold. And the highly porous structure is responsible for cell engraftment [13]. It suggests that the topological structure and modification of biomaterials are significant for stem cell activities. Poly(ethylene glycol) (PEG) is another biodegradable vehicle for stem cell delivery. PEG-based hydrogels associated with ROS-degradable poly(thioketal) (PTK) polymers scavenge free radicals and protect encapsulated MSCs from reactive oxygen species (ROS). And they have special properties of mechanics [127]. In addition, it has been found that adipose-derived MSC (Ad-MSC) paracrine on the electrospun fibers is more apparent than that of those cultured on microplates. It also produces intensive anti-inflammatory and proangiogenic responses, including recruiting macrophages and inducing macrophage polarization to the prohealing phenotype. These properties will be reversed if the NF-*κ*B signaling pathway is inhibited. And the findings demonstrate that the fibrous topography of scaffolds is responsible for regulating the paracrine function. The aligned electrospun fibers show more obvious effects [128]. Nonetheless, considering the productions of these polymers are complex and valuable, they are difficult to apply clinically [129].

The clinical demands of stem cell-based biomaterials are required to have excellent mechanical and biological properties. They are able to promote cell viability, migration, adhesion, differentiation, paracrine function, and the mature structural form in clinical treatment. The physical form of the scaffold can adjust to the morphogenesis of the damaged tissue [119]. It potentially relies on the pore sizes and the modifications of the biomaterials. Importantly, vascularization and anti-inflammatory actions of the regeneration process with multiple growth factors play essential roles. Besides, the economic conditions must be considered in clinical trials. At present, multiple biomaterials for tissue regeneration are still restricted and the clinical transformation of theses biomaterials should be explored further.

## 6. Conclusions

In this review, we describe the step-by-step integration of the organic matrix and minerals following the periodic patterns. The accurate spatial architectures with specific periodicities mightily interact with bone matrix dynamics. The disordered syntheses, assemblies, orientations, and degradations will disrupt bone remodeling and lead to bone defects such as bone fracture and tumors. According to the active remodeling process of ECM, therapeutic strategies target the specific ECM components and the most effective curing time. And more clinical trials apply agonists or inhibitors to target ECM components at an optimal timing, like the timing of MMP inhibitor administration at the different stages of diseases. However, a lot of improvements are required for clinical transformation and personalized medicine. Further researches should systematically complete spatial-temporal patterns and explore the intrinsic mechanisms of ECM remodeling.

Additionally, ECM plays a crucial role in regulating the niches of MSCs, which are responsible for tissue regeneration. In regenerative medicine, MSCs within specific microenvironments can achieve self-renewal and self-repair in normal physiological conditions. The MSC-driven secretome serves as the major promoter of the regenerative potential, including multidirectional differentiation, immunoregulation, and angiogenesis. For clinical application, growing biomaterials specifically interact with stem cells through controlling biomaterial chemical modification, structure, and the addition of biological molecules. Importantly, the limitations of those scaffolds should be improved for clinical transformation and feasibility. Furthermore, the interior connections of ECM and stem cells need to be more extensively explored, which may promote the multipotency of MSC-based scaffolds for multiple tissue repair.

## References

[1] Paiva K. B. S., Granjeiro J. M. (2017). Matrix metalloproteinases in bone resorption, remodeling, and repair. *Progress in Molecular Biology and Translational Science*.

[2] Mansour A., Mezour M. A., Badran Z., Tamimi F. (2017). Extracellular matrices for bone regeneration: a literature review. *Tissue Engineering*.

[3] Bonnans C., Chou J., Werb Z. (2014). Remodelling the extracellular matrix in development and disease. *Nature Reviews Molecular Cell Biology*.

[4] Orgel J. P., Irving T. C., Miller A., Wess T. J. (2006). Microfibrillar structure of type I collagen in situ. *Proceedings of the National Academy of Sciences*.

[5] Blair H. C., Larrouture Q. C., Tourkova I. L. (2018). Support of bone mineral deposition by regulation of pH. *American Journal of Physiology Cell Physiology*.

[6] Burris T. P. (2020). Clock regulation of protein secretion. *Nature Cell Biology*.

[7] Kc R., Li X., Voigt R. M. (2015). Environmental disruption of circadian rhythm predisposes mice to osteoarthritis-like changes in knee joint. *Journal of Cellular Physiology*.

[8] Wu M., Chen G., Li Y. P. (2016). TGF-*β* and BMP signaling in osteoblast, skeletal development, and bone formation, homeostasis and disease. *Bone research*.

[9] Man K., Loudon A., Chawla A. (2016). Immunity around the clock. *Science*.

[10] Parekkadan B., Milwid J. M. (2010). Mesenchymal stem cells as therapeutics. *Annual Review of Biomedical Engineering*.

[11] Ballini A., Boccaccio A., Saini R., Van Pham P., Tatullo M. (2017). Dental-derived stem cells and their secretome and interactions with bioscaffolds/biomaterials in regenerative medicine: from the in vitro research to translational applications. *Stem Cells International*.

[12] Hiew V. V., Simat S. F. B., Teoh P. L. (2018). The advancement of biomaterials in regulating stem cell fate. *Stem Cell Reviews and Reports*.

[13] Tatullo M., Spagnuolo G., Codispoti B. (2019). PLA-based mineral-doped scaffolds seeded with human periapical cyst-derived MSCs: a promising tool for regenerative healing in dentistry. *Materials*.

[14] Zheng C., Chen J., Liu S., Jin Y. (2019). Stem cell-based bone and dental regeneration: a view of microenvironmental modulation. *International Journal of Oral Science*.

[15] Unal M., Creecy A., Nyman J. S. (2018). The role of matrix composition in the mechanical behavior of bone. *Current Osteoporosis Reports*.

[16] Forlino A., Cabral W. A., Barnes A. M., Marini J. C. (2011). New perspectives on osteogenesis imperfecta. *Nature Reviews. Endocrinology*.

[17] Terajima M., Perdivara I., Sricholpech M. (2014). Glycosylation and Cross-linking in Bone Type I Collagen. *The Journal of Biological Chemistry*.

[18] Garnero P. (2015). The role of collagen organization on the properties of bone. *Calcified Tissue International*.

[19] Xu Z., Zhao W., Wang Z., Yang Y., Sahai N. (2018). Structure analysis of collagen fibril at atomic-level resolution and its implications for intra-fibrillar transport in bone biomineralization. *Physical Chemistry Chemical Physics*.

[20] Simon P., Grüner D., Worch H. (2018). First evidence of octacalcium phosphate@osteocalcin nanocomplex as skeletal bone component directing collagen triple-helix nanofibril mineralization. *Scientific Reports*.

[21] Golub E. E. (2011). Biomineralization and matrix vesicles in biology and pathology. *Seminars in Immunopathology*.

[22] Anderson H. C., Sipe J. B., Hessle L. (2004). Impaired calcification around matrix vesicles of growth plate and bone in alkaline phosphatase-deficient mice. *The American Journal of Pathology*.

[23] Pandya M., Rosene L., Farquharson C., Millán J. L., Diekwisch T. G. H. (2017). Intravesicular phosphatase PHOSPHO1 function in enamel mineralization and prism formation. *Frontiers in Physiology*.

[24] Damek-Poprawa M., Golub E., Otis L., Harrison G., Phillips C., Boesze-Battaglia K. (2006). Chondrocytes utilize a cholesterol-dependent lipid translocator to externalize phosphatidylserine. *Biochemistry*.

[25] Wu L. N., Genge B. R., Wuthier R. E. (2008). Analysis and Molecular Modeling of the Formation, Structure, and Activity of the Phosphatidylserine-Calcium-Phosphate Complex Associated with Biomineralization∗. *The Journal of Biological Chemistry*.

[26] Burton B., Gaspar A., Josey D., Tupy J., Grynpas M. D., Willett T. L. (2014). Bone embrittlement and collagen modifications due to high-dose gamma- irradiation sterilization. *Bone*.

[27] Makowski A. J., Uppuganti S., Wadeer S. A. (2014). The loss of activating transcription factor 4 (ATF4) reduces bone toughness and fracture toughness. *Bone*.

[28] Chang J., Garva R., Pickard A. (2020). Circadian control of the secretory pathway maintains collagen homeostasis. *Nature Cell Biology*.

[29] Pickard A., Chang J., Alachkar N. (2019). Preservation of circadian rhythms by the protein folding chaperone, BiP. *The FASEB Journal*.

[30] Boot-Handford R. P., Briggs M. D. (2010). The unfolded protein response and its relevance to connective tissue diseases. *Cell and Tissue Research*.

[31] Marini J. C., Forlino A., Cabral W. A. (2007). Consortium for osteogenesis imperfecta mutations in the helical domain of type I collagen: regions rich in lethal mutations align with collagen binding sites for integrins and proteoglycans. *Human Mutation*.

[32] Swanson C. M., Kohrt W. M., Wolfe P. (2019). Rapid suppression of bone formation marker in response to sleep restriction and circadian disruption in men. *Osteoporosis International*.

[33] Swanson C. M., Shea S. A., Kohrt W. M. (2020). Sleep restriction with circadian disruption negatively alter bone turnover markers in women. *The Journal of Clinical Endocrinology and Metabolism*.

[34] Redmond J., Jarjou L. M., Zhou B., Prentice A., Schoenmakers I. (2014). Ethnic differences in calcium, phosphate and bone metabolism. *The Proceedings of the Nutrition Society*.

[35] Lacruz R. S., Hacia J. G., Bromage T. G. (2012). The circadian clock modulates enamel development. *Journal of Biological Rhythms*.

[36] Lacruz R. S., Nanci A., Kurtz I., Wright J. T., Paine M. L. (2010). Regulation of pH during amelogenesis. *Calcified Tissue International*.

[37] Ohtsuka-Isoya M., Hayashi H., Shinoda H. (2001). Effect of suprachiasmatic nucleus lesion on circadian dentin increment in rats. *American Journal of Physiology-Regulatory, Integrative and Comparative Physiology*.

[38] Papakyrikos A. M., Arora M., Austin C. (2020). Biological clocks and incremental growth line formation in dentine. *Journal of Anatomy*.

[39] Ohtsuka M., Saeki S., Igarashi K., Shinoda H. (1998). Circadian rhythms in the incorporation and secretion of ^3^H-proline by odontoblasts in relation to incremental lines in rat dentin. *Journal of Dental Research*.

[40] Moya A., Tambutté S., Tambutté E., Zoccola D., Caminiti N., Allemand D. (2006). Study of calcification during a daily cycle of the coralStylophora pistillata: implications for 'light-enhanced calcification'. *The Journal of Experimental Biology*.

[41] Olde Loohuis N. F. M., Martens G. J. M., van Bokhoven H., Kaplan B. B., Homberg J. R., Aschrafi A. (2017). Altered expression of circadian rhythm and extracellular matrix genes in the medial prefrontal cortex of a valproic acid rat model of autism. *Progress in Neuro-Psychopharmacology & Biological Psychiatry*.

[42] Li S. K., Banerjee J., Jang C., Sehgal A., Stone R. A., Civan M. M. (2015). Temperature oscillations drive cycles in the activity of MMP-2,9 secreted by a human trabecular meshwork cell line. *Investigative Ophthalmology & Visual Science*.

[43] Nishijima T., Kawakami M., Kita I. (2015). A bout of treadmill exercise increases matrix metalloproteinase-9 activity in the rat hippocampus. *Neuroscience Letters*.

[44] Gao Y., Xiao Q., Ma H. (2010). LKB1 inhibits lung cancer progression through lysyl oxidase and extracellular matrix remodeling. *Proceedings of the National Academy of Sciences*.

[45] Paszek M. J., Zahir N., Johnson K. R. (2005). Tensional homeostasis and the malignant phenotype. *Cancer Cell*.

[46] Levental K. R., Yu H., Kass L. (2009). Matrix crosslinking forces tumor progression by enhancing integrin signaling. *Cell*.

[47] Kessenbrock K., Plaks V., Werb Z. (2010). Matrix metalloproteinases: regulators of the tumor microenvironment. *Cell*.

[48] Boyd N. F., Martin L. J., Yaffe M. J., Minkin S. (2011). Mammographic density and breast cancer risk: current understanding and future prospects. *Breast Cancer Research*.

[49] Fonseca H., Moreira-Gonçalves D., Coriolano H. J., Duarte J. A. (2014). Bone quality: the determinants of bone strength and fragility. *Sports Medicine*.

[50] de Melo Pereira D., Habibovic P. (2018). Biomineralization-inspired material design for bone regeneration. *Advanced Healthcare Materials*.

[51] Jung G. S., Buehler M. J. (2017). Multiscale modeling of muscular-skeletal systems. *Annual Review of Biomedical Engineering*.

[52] Reznikov N., Shahar R., Weiner S. (2014). Bone hierarchical structure in three dimensions. *Acta Biomaterialia*.

[53] George A., Veis A. (2008). Phosphorylated proteins and control over apatite nucleation, crystal growth, and inhibition. *Chemical Reviews*.

[54] Mahamid J., Aichmayer B., Shimoni E. (2010). Mapping amorphous calcium phosphate transformation into crystalline mineral from the cell to the bone in zebrafish fin rays. *Proceedings of the National Academy of Sciences*.

[55] Nudelman F., Pieterse K., George A. (2010). The role of collagen in bone apatite formation in the presence of hydroxyapatite nucleation inhibitors. *Nature Materials*.

[56] Kim D., Lee B., Thomopoulos S., Jun Y. S. (2018). The role of confined collagen geometry in decreasing nucleation energy barriers to intrafibrillar mineralization. *Nature Communications*.

[57] Xu Y., Nudelman F., Eren E. D. (2020). Intermolecular channels direct crystal orientation in mineralized collagen. *Nature Communications*.

[58] Chen L., Jacquet R., Lowder E., Landis W. J. (2015). Refinement of collagen-mineral interaction: a possible role for osteocalcin in apatite crystal nucleation, growth and development. *Bone*.

[59] Reznikov N., Bilton M., Lari L., Stevens M. M., Kröger R. (2018). Fractal-like hierarchical organization of bone begins at the nanoscale. *Science*.

[60] Wang Y., Von Euw S., Fernandes F. M. (2013). Water-mediated structuring of bone apatite. *Nature Materials*.

[61] Wu Y., Ackerman J. L., Kim H. M., Rey C., Barroug A., Glimcher M. J. (2002). Nuclear magnetic resonance spin-spin relaxation of the crystals of bone, dental enamel, and synthetic hydroxyapatites. *Journal of Bone and Mineral Research: the Official Journal of the American Society for Bone and Mineral Research*.

[62] Liu Y., Kim Y. K., Dai L. (2011). Hierarchical and non-hierarchical mineralisation of collagen. *Biomaterials*.

[63] Ziv V., Wagner H. D., Weiner S. (1996). Microstructure-microhardness relations in parallel-fibered and lamellar bone. *Bone*.

[64] Hynes R. O. (2009). The extracellular matrix: not just pretty fibrils. *Science*.

[65] Meng X. M., Nikolic-Paterson D. J., Lan H. Y. (2016). TGF-*β*: the master regulator of fibrosis. *Nature Reviews Nephrology*.

[66] Kahai S., Vary C. P., Gao Y., Seth A. (2004). Collagen, type V, *α*1 (COL5A1) is regulated by TGF-*β* in osteoblasts. *Matrix Biology*.

[67] Crane J. L., Xian L., Cao X. (2016). Role of TGF-*β* signaling in coupling bone remodeling. *Methods in Molecular Biology*.

[68] Dennler S., Pendaries V., Tacheau C., Costas M. A., Mauviel A., Verrecchia F. (2005). The steroid receptor co-activator-1 (SRC-1) potentiates TGF- *β* /Smad signaling: role of p300/CBP. *Oncogene*.

[69] Kushioka J., Kaito T., Okada R. (2020). A novel negative regulatory mechanism of Smurf2 in BMP/Smad signaling in bone. *Bone Research*.

[70] Kang J. S., Alliston T., Delston R., Derynck R. (2005). Repression of Runx2 function by TGF-*β* through recruitment of class II histone deacetylases by Smad3. *The EMBO Journal*.

[71] Sowa H., Kaji H., Yamaguchi T., Sugimoto T., Chihara K. (2002). Activations of ERK1/2 and JNK by Transforming Growth Factor *β* Negatively Regulate Smad3-induced Alkaline Phosphatase Activity and Mineralization in Mouse Osteoblastic Cells∗. *The Journal of Biological Chemistry*.

[72] Sun X., Xie Z., Ma Y. (2018). TGF-*β* inhibits osteogenesis by upregulating the expression of ubiquitin ligase SMURF1 via MAPK-ERK signaling. *Journal of Cellular Physiology*.

[73] Dudek M., Gossan N., Yang N. (2016). The chondrocyte clock gene Bmal1 controls cartilage homeostasis and integrity. *The Journal of Clinical Investigation*.

[74] Dong C., Gongora R., Sosulski M. L., Luo F., Sanchez C. G. (2016). Regulation of transforming growth factor-beta1 (TGF-*β*1)-induced pro-fibrotic activities by circadian clock gene BMAL1. *Respiratory Research*.

[75] Shen B., Wei A., Whittaker S. (2009). The role of BMP-7 in chondrogenic and osteogenic differentiation of human bone marrow multipotent mesenchymal stromal cells in vitro. *Journal of Cellular Biochemistry*.

[76] Zuzarte-Luís V., Montero J. A., Rodriguez-León J., Merino R., Rodríguez-Rey J. C., Hurlé J. M. (2004). A new role for BMP5 during limb development acting through the synergic activation of Smad and MAPK pathways. *Developmental Biology*.

[77] Arndt S., Karrer S., Hellerbrand C., Bosserhoff A. K. (2019). Bone morphogenetic protein-6 inhibits fibrogenesis in scleroderma offering treatment options for fibrotic skin disease. *The Journal of Investigative Dermatology*.

[78] Huang Z., Ren P. G., Ma T., Smith R. L., Goodman S. B. (2010). Modulating osteogenesis of mesenchymal stem cells by modifying growth factor availability. *Cytokine*.

[79] Noël D., Gazit D., Bouquet C. (2004). Short-term BMP-2 expression is sufficient for in vivo osteochondral differentiation of mesenchymal stem cells. *Stem Cells*.

[80] Gu K., Zhang L., Jin T., Rutherford R. B. (2004). Identification of potential modifiers of Runx2/Cbfa1 activity in C2C12 cells in response to bone morphogenetic protein-7. *Cells, Tissues, Organs*.

[81] Witt-Enderby P. A., Slater J. P., Johnson N. A. (2012). Effects on bone by the light/dark cycle and chronic treatment with melatonin and/or hormone replacement therapy in intact female mice. *Journal of Pineal Research*.

[82] Min H. Y., Kim K. M., Wee G., Kim E. J., Jang W. G. (2016). Bmal1 induces osteoblast differentiation via regulation of BMP2 expression in MC3T3-E1 cells. *Life Sciences*.

[83] Thampatty B. P., Li H., Im H. J., Wang J. H. (2007). EP_4_ receptor regulates collagen type-I, MMP-1, and MMP-3 gene expression in human tendon fibroblasts in response to IL-1*β* treatment. *Gene*.

[84] Li S., Lu A., Li B., Wang Y. (2004). Circadian rhythms on hypothalamic-pituitary-adrenal axis hormones and cytokines of collagen induced arthritis in rats. *Journal of Autoimmunity*.

[85] Nieto N. (2006). Oxidative-stress and IL-6 mediate the fibrogenic effects of rodent Kupffer cells on stellate cells. *Hepatology*.

[86] McGregor N. E., Murat M., Elango J. (2019). IL-6 exhibits both *cis* \- and *trans* -signaling in osteocytes and osteoblasts, but only *trans* -signaling promotes bone formation and osteoclastogenesis. *The Journal of Biological Chemistry*.

[87] Silver A. C., Arjona A., Walker W. E., Fikrig E. (2012). The circadian clock controls toll-like receptor 9-mediated innate and adaptive immunity. *Immunity*.

[88] Gibbs J. E., Blaikley J., Beesley S. (2012). The nuclear receptor REV-ERB mediates circadian regulation of innate immunity through selective regulation of inflammatory cytokines. *Proceedings of the National Academy of Sciences*.

[89] Deng Z., Hu W., Ai H., Chen Y., Dong S. (2021). The dramatic role of IFN family in aberrant inflammatory osteolysis. *Current Gene Therapy*.

[90] Scheiermann C., Kunisaki Y., Frenette P. S. (2013). Circadian control of the immune system. *Nature Reviews Immunology*.

[91] Ohgushi H. (2014). Osteogenically differentiated mesenchymal stem cells and ceramics for bone tissue engineering. *Expert Opinion on Biological Therapy*.

[92] Sui B. D., Hu C. H., Zheng C. X., Jin Y. (2016). Microenvironmental views on mesenchymal stem cell differentiation in aging. *Journal of Dental Research*.

[93] Ferreira J. R., Teixeira G. Q., Santos S. G., Barbosa M. A., Almeida-Porada G., Gonçalves R. M. (2018). Mesenchymal stromal cell secretome: influencing therapeutic potential by cellular pre-conditioning. *Frontiers in Immunology*.

[94] Liu X., Duan B., Cheng Z. (2011). SDF-1/CXCR4 axis modulates bone marrow mesenchymal stem cell apoptosis, migration and cytokine secretion. *Protein & Cell*.

[95] Cheng Z., Ou L., Zhou X. (2008). Targeted migration of mesenchymal stem cells modified with CXCR4 gene to infarcted myocardium improves cardiac performance. *Molecular Therapy*.

[96] Zou C., Luo Q., Qin J. (2013). Osteopontin promotes mesenchymal stem cell migration and lessens cell stiffness via integrin *β*1, FAK, and ERK pathways. *Cell Biochemistry and Biophysics*.

[97] Fu X., Liu G., Halim A., Ju Y., Luo Q., Song A. G. (2019). Mesenchymal stem cell migration and tissue repair. *Cell*.

[98] Chen G., Deng C., Li Y. P. (2012). TGF-*β* and BMP signaling in osteoblast differentiation and bone formation. *International Journal of Biological Sciences*.

[99] Eguchi K., Akiba Y., Akiba N., Nagasawa M., Cooper L. F., Uoshima K. (2018). Insulin-like growth factor binding protein-3 suppresses osteoblast differentiation via bone morphogenetic protein-2. *Biochemical and Biophysical Research Communications*.

[100] Ge C., Yang Q., Zhao G., Yu H., Kirkwood K. L., Franceschi R. T. (2012). Interactions between extracellular signal-regulated kinase 1/2 and p 38 MAP kinase pathways in the control of RUNX2 phosphorylation and transcriptional activity. *Journal of Bone and Mineral Research: the Official Journal of the American Society for Bone and Mineral Research*.

[101] Ortuño M. J., Ruiz-Gaspà S., Rodríguez-Carballo E. (2010). p38 Regulates Expression of Osteoblast-specific Genes by Phosphorylation of Osterix∗. *The Journal of Biological Chemistry*.

[102] Yang X., Matsuda K., Bialek P. (2004). ATF4 Is a Substrate of RSK2 and an Essential Regulator of Osteoblast Biology: Implication for Coffin-Lowry Syndrome. *Cell*.

[103] Beresford J. N., Bennett J. H., Devlin C., Leboy P. S., Owen M. E. (1992). Evidence for an inverse relationship between the differentiation of adipocytic and osteogenic cells in rat marrow stromal cell cultures. *Journal of Cell Science*.

[104] Selvasandran K., Makhoul G., Jaiswal P. K. (2018). A tumor necrosis factor-*α* and hypoxia-induced secretome therapy for myocardial repair. *The Annals of Thoracic Surgery*.

[105] Nakamura Y., Ishikawa H., Kawai K., Tabata Y., Suzuki S. (2013). Enhanced wound healing by topical administration of mesenchymal stem cells transfected with stromal cell-derived factor-1. *Biomaterials*.

[106] Rani S., Ryan A. E., Griffin M. D., Ritter T. (2015). Mesenchymal stem cell-derived extracellular vesicles: toward cell-free therapeutic applications. *Molecular Therapy*.

[107] Tögel F., Hu Z., Weiss K., Isaac J., Lange C., Westenfelder C. (2005). Administered mesenchymal stem cells protect against ischemic acute renal failure through differentiation-independent mechanisms. *American Journal of Physiology. Renal Physiology*.

[108] Maggini J., Mirkin G., Bognanni I. (2010). Mouse bone marrow-derived mesenchymal stromal cells turn activated macrophages into a regulatory-like profile. *PLoS One*.

[109] Aggarwal S., Pittenger M. F. (2005). Human mesenchymal stem cells modulate allogeneic immune cell responses. *Blood*.

[110] Gieseke F., Böhringer J., Bussolari R., Dominici M., Handgretinger R., Müller I. (2010). Human multipotent mesenchymal stromal cells use galectin-1 to inhibit immune effector cells. *Blood*.

[111] Qiu G., Zheng G., Ge M. (2019). Functional proteins of mesenchymal stem cell-derived extracellular vesicles. *Stem Cell Research & Therapy*.

[112] Simpson R. J., Jensen S. S., Lim J. W. (2008). Proteomic profiling of exosomes: current perspectives. *Proteomics*.

[113] Kim H. S., Choi D. Y., Yun S. J. (2012). Proteomic analysis of microvesicles derived from human mesenchymal stem cells. *Journal of Proteome Research*.

[114] Lai R. C., Tan S. S., Teh B. J. (2012). Proteolytic potential of the MSC exosome proteome: implications for an exosome-mediated delivery of therapeutic proteasome. *International Journal of Proteomics*.

[115] Feng Y., Huang W., Wani M., Yu X., Ashraf M. (2014). Ischemic preconditioning potentiates the protective effect of stem cells through secretion of exosomes by targeting Mecp2 via miR-22. *PLoS One*.

[116] Toh W. S., Lai R. C., Zhang B., Lim S. K. (2018). MSC exosome works through a protein-based mechanism of action. *Biochemical Society Transactions*.

[117] Wobma H. M., Tamargo M. A., Goeta S., Brown L. M., Duran-Struuck R., Vunjak-Novakovic G. (2018). The influence of hypoxia and IFN-*γ* on the proteome and metabolome of therapeutic mesenchymal stem cells. *Biomaterials*.

[118] Lambricht L., De Berdt P., Vanacker J. (2014). The type and composition of alginate and hyaluronic-based hydrogels influence the viability of stem cells of the apical papilla. *Dental materials: official publication of the Academy of Dental Materials*.

[119] Raddall G., Mello I., Leung B. M. (2019). Biomaterials and scaffold design strategies for regenerative endodontic therapy. *Frontiers in Bioengineering and Biotechnology*.

[120] Busilacchi A., Gigante A., Mattioli-Belmonte M., Manzotti S., Muzzarelli R. A. (2013). Chitosan stabilizes platelet growth factors and modulates stem cell differentiation toward tissue regeneration. *Carbohydrate Polymers*.

[121] Xia W., Li H., Wang Z. (2011). Human platelet lysate supportsex vivoexpansion and enhances osteogenic differentiation of human bone marrow-derived mesenchymal stem cells. *Cell Biology International*.

[122] Chang B., Ahuja N., Ma C., Liu X. (2017). Injectable scaffolds: preparation and application in dental and craniofacial regeneration.. *Materials Science and Engineering: R: Reports*.

[123] Aulino P., Costa A., Chiaravalloti E. (2015). Muscle extracellular matrix scaffold is a multipotent environment. *International Journal of Medical Sciences*.

[124] Lee I. C., Lee Y. T., Yu B. Y., Lai J. Y., Young T. H. (2009). The behavior of mesenchymal stem cells on micropatterned PLLA membranes. *Journal of Biomedical Materials Research. Part A*.

[125] Chung C., Burdick J. A. (2008). Engineering cartilage tissue. *Advanced Drug Delivery Reviews*.

[126] Xu Z., Liu P., Li H., Zhang M., Wu Q. (2020). In vitro study on electrospun lecithin-based poly (L-lactic acid) scaffolds and their biocompatibility. *Journal of Biomaterials Science. Polymer Edition*.

[127] Martin J. R., Patil P., Yu F., Gupta M. K., Duvall C. L. (2020). Enhanced stem cell retention and antioxidative protection with injectable, ROS-degradable PEG hydrogels. *Biomaterials*.

[128] Su N., Gao P. L., Wang K., Wang J. Y., Zhong Y., Luo Y. (2017). Fibrous scaffolds potentiate the paracrine function of mesenchymal stem cells: a new dimension in cell-material interaction. *Biomaterials*.

[129] Wang W., Dang M., Zhang Z. (2016). Dentin regeneration by stem cells of apical papilla on injectable nanofibrous microspheres and stimulated by controlled BMP-2 release. *Acta Biomaterialia*.

